# *Acinetobacter baumannii* Septicemia in a Recipient of an Allogeneic Hematopoietic Stem Cell Transplantation

**DOI:** 10.1155/2012/646195

**Published:** 2012-08-07

**Authors:** Khalid Ahmed Al-Anazi, Baha Abdalhamid, Zeyad Alshibani, Khalid Awad, Abdullah Alzayed, Hoda Hassan, Mohammed Alsayiegh

**Affiliations:** ^1^Section of Adult Hematology and Hematopoietic, Stem Cell Transplant, Oncology Center, King Fahad Specialist Hospital, P.O. Box 15215, Dammam 31444, Saudi Arabia; ^2^Section of Microbiology, Department of Pathology and Laboratory Medicine, King Fahad Specialist Hospital, P.O. Box 15215, Dammam 31444, Saudi Arabia

## Abstract

*Acinetobacter baumannii* is a gram-negative, nonfermentative coccobacillus that causes infections in immunocompromised and chronically ill patients and is associated with multidrug resistance. Two days before receiving her nonmyeloablative stem cell allograft, a patient with acute myeloid leukemia developed *Acinetobacter baumannii* bacteremia that caused septic shock which was successfully treated with imipenem and removal of the central venous catheter. To our knowledge, this is the first report of *Acinetobacter baumannii* septicemia in a hematopietic stem cell transplantation recipient.

## 1. Introduction

Nonmyeloablative (NMA) hematopoietic stem cell transplantation (HSCT) has been explored in patients with malignant hematological disorders in order to minimize treatment-related toxicity of HSCT [1]. The incidence of bacterial, fungal, and viral infections is lower in patients subjected to NMA-HSCT than in patients undergoing standard or conventional allogeneic HSCT [1, 2]. The lower overall risk of infectious complications in patients receiving NMA-HSCT is due to the shorter duration of neutropenia and the lower incidence of graft versus host disease (GVHD) in comparison to conventional allografting [2]. The risk factors of infectious complications related to NMA-HSCT include old age, matched related donor grafts, and postengraftment period as most bacterial infections develop after engraftment [2].

Inadequate initial treatment in serious infections leads to increased mortality. However, achieving adequate therapy is increasingly difficult because of increasing prevalence of multidrug resistant (MDR) pathogens [3]. The carbapenems are potent, safe, efficacious, and broad-spectrum antibiotics in the treatment of serious bacterial infections. They provide better gram-negative (GN) coverage than other *β*-lactams and are stable against extended spectrum *β*-lactamases (ESBL) and Amp C *β*-lactamases, making them effective in the treatment of many MDR bacteria. The newly approved carbapenem, doripenem may help preserve the utility of the carbapenem class of antibiotics [3].

The spectrum of isolates among HSCT patients having febrile neutropenia (FN) appears to be shifting towards GN microorganisms. Due to increasing levels of drug resistance among the isolates, a glycopeptide in combination with a carbapenem would be prudent choice as empiric therapy in high-risk individuals [4]. In patients with acute leukemia and HSCT with FN, microbiologically documented infections occur in 30% of FN episodes, bacteremia develop in 26% of episodes of FN and GN isolates account for 55.7% of documented infections. Cefoperazone-sulbactam combination has the highest in vitro efficacy against GN rods and carbapenem resistance is most prevalent among *Acinetobacter* spp. [5]. The overall mortality rate (MR) due to FN is 8%, and mortality is associated with a nadir leucocyte count of < 200 /*μ*L and an abnormal chest radiograph. In high-risk FN patients, a frontline regimen containing cefoperazone sulbactam could restrict the use of imipenem and result in an acceptable mortality [5].

## 2. Case Report

A 58-year-old Saudi female was diagnosed to have acute myeloid leukemia (AML) at King Fahad Specialist Hospital (KFSH) in Dammam, Saudi Arabia in late October 2010. She presented with fever, mucosal bleeding, and anemic manifestations. Her physical examination at presentation showed no external palpable lymphadenopathy or palpable abdominal organomegaly. Her chest, cardiovascular, and neurological examinations were normal. Complete blood count (CBC) showed pancytopenia and blood film revealed 40% myeloblasts. Bone marrow examination (BME) was consistent with AML with negative cytogenetics. Renal, hepatic, and coagulation profiles were all within normal limits. Chest radiography, electrocardiogram, and echocardiogram were also normal. After confirming the diagnosis of AML, the patient was commenced on an induction course of chemotherapy (3 + 7 protocol) composed of idarubicin and cytosine arabinoside. As there was no response to the induction treatment, MEC (mitoxantrone, etoposide, and cytosine arabinoside) salvage chemotherapy was given, but unfortunately the patient showed no response to it. Following the 2 cycles of chemotherapy given, the patient developed several FN episodes that were controlled with empirical antimicrobials. Due to her old age, refractoriness of her AML to 2 lines of intensive chemotherapy and the infectious complications encountered, it was decided to shift the patient to palliative chemotherapy in the form of low-dose Ara-C (cytosine arabinoside) given subcutaneously for 10 days. Following the third line of chemotherapy, the patient reported to the out patient clinic on 13/02/2011 with no complaints, her physical examination revealed no new abnormality and her blood counts were normal. A new blood film showed no blasts and a repeat BME showed evidence of first complete remission (CR) of her AML. As an identical sibling donor for allogeneic HSCT had already been identified, it was decided to offer her a NMA allogeneic HSCT. Meanwhile, she received a second cycle of low-dose Ara-C to keep her AML in remission.

On 14/03/2011; the patient was readmitted for allogeneic HSCT in a stable clinical condition. Her blood counts were normal, blood film showed no blasts, and her pre-HSCT BME showed evidence of continued CR. The transplant conditioning protocol was composed of 1 session of total body irradiation and 3 doses of fludarabine. She received phenoxymethylpenicillin, fluconazole, and acyclovir as infection prophylaxis and cyclosporine-A and mycophenolate mofetil as GVHD prophylaxis.

Two days prior to her allograft, the patient became highly febrile with no clinical focus of infection. Two sets of standard blood cultures (aerobic and anaerobic bottles) were drawn peripherally and centrally, and the patient was commenced on intravenous (IV) piperacillin tazobactam. Blood cultures were processed with the BACTEC 9240 system and bacterial growth was detected in aerobic bottles of the two sets on day 2 of incubation at 37°C. Gram stain revealed GN coccobacilli and the organism was subcultured on blood agar, MacConkey agar, and chocolate agar and incubated at 37°C in 5% CO_2_. The organism grew on all culture types and the colonies were nonlactose fermenters and oxidase negative. The organism was identified as *Acinetobacter baumannii *(*A. baumannii*) complex using the VITEK2 (bioMerieux, Marcy, France). The Microseq 500 16S rDNA bacterial identification kit (Applied Biosystem, Foster City, CA, USA), composed of polymerase chain reaction (PCR), cycle sequencing modules, analysis software, and a 500-bp 16S rDNA library of bacterial nucleic acid sequences was used for microbial identification as recommended by the manufacturer. The nucleotide sequence of the PCR product of the 16S rRNA gene revealed that it was identical to that of *A. baumannii*. Antimicrobial susceptibility testing of the isolate was carried out using the GN93 card of the VITEK2 and disk diffusion. Minimum inhibitory concentrations (MICs) were interpreted using Clinical Laboratory Standards Institute (CLSI) guidelines. *Escherichia coli* ATCC 25922 and *Pseudomonas aeruginosa* ATCC 27853 were used as quality controls. The organism was susceptible to cefepime, ciprofloxacin, gentamicin, imipenem, and meropenem and resistant to piperacillin tazobactam.

The patient remained hemodynamically unstable as she continued to be highly febrile and her diastolic blood pressure dropped by 20 mmHg, so IV fluid rate was increased and piperacillin tazobactam was replaced by imipenem and vancomycin. One day prior to her allograft, blood cultures taken from the central venous catheter (CVC) were reported to be positive for *A. baumannii*. Consequently, the CVC was removed, imipenem and vancomycin were continued.

On 21/03/2011, the patient became afebrile, normotensive and hemodynamically stable, and this allowed infusion of the harvested stem cells without complications. The source of stem cells was peripheral blood and CD 34+ stem cell dose was 4.57 × 10^6^/kg body weight. Five days following her allograft, the patient developed grade I mucositis that was controlled by analgesics as needed and antiseptic mouth care. She engrafted her leucocytes on day +21 HSCT. Her platelet count never dropped below 82 × 10^9^/L in the early posttransplant period and it exceeded 100 × 10^9^/L on day +14 HSCT. After the recovery of her blood counts and the control of her *Acinetobacter* sepsis, the patient was discharged on 20/1/2011 on: cyclosporine-A, pantoprazole, phenoxymethylpenicillin, fluconazole, and acyclovir. Thereafter, the patient had regular followup at the HSCT outpatient clinic.

## 3. Discussion

*A. baumannii* are GN, nonfermentative, coccobacilli that are widely distributed in nature and that are characterized by frequent MDR due to multiple mechanisms [6–8]. The genus *Acinetobacter *comprises 30 different species. * A. baumannii,* the genotypically related *Acinetobacter *genomic species 3 and *Acinetobacter* genomic species 13 TU are the most pathogenic types in humans [8]. The sources of *A. baumannii* infection include wounds, burns, intravascular and urinary catheters and blood in addition to gastrointestinal, respiratory, and urinary tracts. At times, no source of infection or bacteremia is found [6, 9–11]. The ability of *A. baumannii* to survive under a wide range of environmental conditions and to persist for extended periods of time on surfaces makes it a frequent cause of infection outbreaks and an endemic infection [12, 13]. Coinfection with other organisms is common and it has been reported in as high as 35% of cases [6].

The risk factors for *A. baumannii* infections, bacteremia, and colonization include burns, surgical wounds, recent major surgical procedures, CVCs, and severe underlying illness particularly critically ill patients and those with hematologic malignancy, prolonged antimicrobial therapy with broad spectrum antibiotics, urinary tract infections and catheterization, respiratory tract colonization, infection and endotracheal intubation, and intestinal colonization in addition to infections due to bacterial translocation [6, 10–12]. The clinical manifestations of *A. baumannii* infection include nonspecific features, transient maculopapular rash, local infections involving wounds, respiratory and gastrointestinal tracts, and infections of catheters. However, such infections may be complicated by extensive necrosis of soft tissues, bacteremia, endocarditis, disseminated intravascular coagulation, systemic infection with progressive clinical course, septic shock, multiorgan failure, and death [6, 9, 10]. Septic shock has been reported in up to 42% of patients having bacteremia and crude MR in patients with bacteremia may reach 70%. However, complicated clinical course and life-threatening complications are more likely to occur in immunocompromised individuals [6, 9, 10, 13, 14].

*A. baumannii-calcoaceticus* complex plays a significant role in health care systems as this serious nosocomial pathogen has the ability to persist in hospital environment and is also capable of rapidly acquiring resistance to broad spectrum antimicrobial agents [15]. The virulence factors of *A. baumannii* are largely unknown. Bacterial factors may be associated with the outcome in patients with *A. baumannii* bacteremia [16]. Recently, *A. baumannii *has emerged as one of the most troublesome pathogens for health care institutions globally as large numbers of nosocomial infections and bacteremia have been reported worldwide [6, 7, 13, 16–19]. Community-acquired infections and bacteremia caused by *A. baumannii* are much less common than nosocomial infections. Patients with pneumonic community acquired *A. baumannii* (CAAB) infections have worse prognosis than nonpneumonic CAAB infections. The development of CAAB infection is associated with the underlying illness or malignancy. *A. baumannii* genomic species is responsible for the majority of cases of CAAB infections. However, accurate species level identification of *Acinetobacter* species is difficult. There is no evidence that clonal spread of *A. baumannii* in the community has resulted in multiple cases of CAAB infections. Most of the previously reported episodes of CAAB infections were caused by pneumonia. Ceftazidime, cefepime, cefpirome, carbapenems, aminoglycosides, and fluoroquinolones are the drugs of choice for the treatment of CAAB infections [18].

*A. baumannii* commonly targets critically ill patients with breaches in skin integrity and airway protection. Hospital acquired pneumonia is the commonest infection caused by *A. baumannii* and it carries the highest rate of mortality. Recently, infections involving skin, soft tissues, bone, and central nervous system have emerged as highly problematic for certain institutions [13]. The risk factors for *A. baumannii* bacteremia include immunosuppression, unscheduled admission to hospital, previous antimicrobial therapy, previous intensive care unit (ICU) sepsis, development of sepsis or septic shock, respiratory failure on admission to ICU, and the use of invasive procedures [17, 20]. The sequential organ failure assessment (SOFA) and the acute physiology and chronic health evaluation (APACHE) II scores determined at the onset of *A. baumannii* bacteremia are reliable risk stratifying tools that predict 14-day and in-hospital mortality [16]. In patients with *A. baumannii* bacteremia it is essential to perform thorough clinical evaluation of the patient to eliminate the possibility of a pseudobacteremia as only 62% of bacteremia in hospitalized patients are considered clinically significant [9, 10, 14]. Approximately 35% of patients with *A. baumannii* bacteremia have polymicrobial bacteremia or other infections and about 42% of patients with bacteremia develop septic shock [6, 10, 14]. Management of *A. baumannii* infections includes removal of vascular or urinary catheters, surgical therapy of the source of infection or bacteremia, and antimicrobial therapy. The drug of choice for treatment of *A. baumannii* infections is not yet established, but the most effective drugs are imipenem, ampicillin-sulbactam, and ceftazidime [6]. However, isolates of *A. baumannii* have shown high levels of antimicrobial resistance, particularly to *β*-lactam agents [11]. Antimicrobial resistance among *Acinetobacter* spp. has substantially increased in the past decade. MDR *A. baumannii* infections carry a high crude MR and have a great impact on health care settings as they are most frequently encountered in severely ill patients [12].

*Acinetobacter* spp. possesses a wide array of *β*-lactamases that hydrolyze and confer resistance to penicillins, cephalosporins, and carbapenems. Carbapenem resistance in *Acinetobacter* spp. has been linked to point mutations in porin channels from the outer membrane. These point mutations alter bacterial targets or functions, thus decreasing the affinity for antimicrobial agents or upregulating cellular functions by producing efflux pumps or other proteins [12]. Antimicrobial agents that are potentially effective against MDR *A. baumannii* strains include carbapenems, aminoglycosides (gentamicin and amikacin), tetracyclines (minocycline and doxycycline), sulbactam, colistin, and combinations of different antimicrobials [6–8]. Thus, treatment options for MDR *Acinetobacter* infections are severely limited and there have been no controlled trials to guide the therapeutic choices. Carbapenems and colistin can be considered the agents of choice for most MDR *Acinetobacter* strains. However, the role of other agents or combination therapy remains unclear [12]. New experimental studies are warranted to evaluate the activity and safety of peptides and other novel antibacterial agents for *A. baumannii* infections [21].

In a European survey, resistance of *A. baumannii* rated 5 among the evolving bacterial resistance to antimicrobial therapy [22]. Development of MDR *A. baumannii* phenotypes is related to the use of carbapenems and third-generation cephalosporins. Also, the risk factors for the development of MDR *A. baumannii* are so diverse that a separate outbreak investigations should be performed in each hospital setting [23]. When the bacterium represents infection rather than colonization, prompt and adequate treatment with antimicrobial agents is required based on the results of in-vitro susceptibility [15]. Although imipenem is the most active agent against *A. baumannii*, resistance to imipenem is becoming increasingly common. As infections caused by strains exhibiting resistance to carbapenems and polymyxins are regularly observed and as strains of *A. baumannii* that are resistant to all known antimicrobials have recently been reported, treatment of *A. baumannii* infections should be carefully considered [7, 9, 13, 19].

Although hospital outbreaks of imipenem-resistant MDR *A. baumannii* (IR-MDR-AB) have been increasingly reported since the early 1990s, carbapenems are still considered the agents of choice for patients with *A. baumannii* infections as MDR *A. baumannii* strains have retained in vitro susceptibility to carbapenems. The emergence of IR-MDR-AB infections has become a world-wide problem and has imposed a grave concern in clinical practice as these organisms are susceptible to few drugs in vitro and this threatens successful therapy of infections caused by* Acinetobacter* spp. The risk factors for IR-MDR-AB infections are previous ICU admission, exposure to third-generation cephalosporins, and use of carbapenems [24, 25]. The time at risk (the period of time at risk for appearance of IR-MDR-AB) is a very important confounding factor to be adjusted because the probability of appearance increases with the length of time [24].

Despite that MDR, *A. baumannii* strains have retained susceptibility to colistin, a recent study showed that administration of colistin does not improve the overall 30 day-in hospital mortality in patients having MDR *A. baumannii* bloodstream infections and that clinical severity of patients may be the only determinant of the outcome of such infections [26]. Tigecycline has also shown considerable, but not consistent, antimicrobial activity against MDR *Acinetobacter* species. Data on the clinical use of tigecycline for the treatment of patients with infections caused by MDR, *Acinetobacter* spp., particularly for ventilator-associated pneumonia or bacteremia, are still scarce and are confounded by the use of tigecycline in combination regimens [8].

Strict infection control measures, adequate use of antimicrobial agents, and appropriate use of invasive procedures could be important preventive measures in decreasing the incidence of *A. baumannii* infections [17]. Development of innovative control strategies and enforcement of aggressive infection control measures should be implemented to limit the spread of these MDR pathogens [15, 23].

Infections caused by *A. baumannii* are associated with considerable attributable mortality and the prognosis of such infections varies considerably [19, 21]. Good prognosis is associated with local infection or trauma, absence of MDR strains, medical conditions other than malignancy or burns as well as prompt and efficient antimicrobial therapy [6, 11]. Poor outcome is associated with: burns or malignancy being the underlying medical condition, age more than 65 years, presence of virulent strain or MDR organism, late or inappropriate antimicrobial therapy, disseminated intravascular coagulation or coagulopathy, bacteremia or septic shock, mechanical ventilation, and rapidly progressive or ultimately fatal illness [6, 10, 11, 14, 16]. Pneumonia, bacteremia, and inappropriate therapy are associated with high mortality [19, 21]. Mortality rates in patients with *A. baumannii* bacteremia range from as low as 5% in general wards to as high as 54 to 63% in ICUs. The overall mortality associated with *A. baumannii* bacteremia ranges between 25 and 54% and depends on the physical condition of the patient. Even in critically ill patients, certain studies have shown that the attributable MR to *A. baumannii* bacteremia varies between 7.8 and 19% [6, 10, 14, 27]. The ultimate outcome of *A. baumannii* infections correlates well with the type of underlying medical illness, polymicrobial bacteremia, and appropriate antibiotic therapy [11].

The patient presented was severely immunocompromised due to having high-risk AML, the repeated cycles of cytotoxic chemotherapy given to control her primary refractory leukemia, the conditioning chemotherapy and radiotherapy given just before HSCT, and the immunosuppressive therapy administered following her stem cell allograft. She also suffered serious infective complications following the induction and salvage cycles of chemotherapy. The CVC was an additional predisposing factor for her *A. baumannii* bacteremia. Administration of imipenem and removal of the CVC, which could have been the source of infection in the absence of a clear clinical focus, were vital to control the bacteremic episode. Prompt intervention with hydration and stat doses of the second-line empirical antibiotics reversed her septic episode and prevented the need for higher level of care in the ICU.

## 4. Conclusion

*Acinetobacter *infections predominantly occur in immunocompromised patients and chronically ill individuals. These infections are variable in severity and clinical presentation but may be lethal. CVC-related infections are common in patients with hematologic malignancy receiving immunosuppressive therapies. Increasing levels of resistance to several classes of antimicrobial agents are a real concern. Early administration of appropriate antimicrobial therapy and removal of the source of infection, if found, are essential to control such infections.
